# Migrastatin Analogues Inhibit Canine Mammary Cancer Cell Migration and Invasion

**DOI:** 10.1371/journal.pone.0076789

**Published:** 2013-10-08

**Authors:** Kinga Majchrzak, Daniele Lo Re, Małgorzata Gajewska, Małgorzata Bulkowska, Agata Homa, Karol Pawłowski, Tomasz Motyl, Paul V. Murphy, Magdalena Król

**Affiliations:** 1 Department of Physiological Sciences, Faculty of Veterinary Medicine, Warsaw University of Life Sciences, Warsaw, Poland; 2 Department of Animal Environment Biology, Faculty of Animal Sciences, Warsaw University of Life Sciences, Warsaw, Poland; 3 School of Chemistry, National University of Ireland, Galway, Ireland; 4 Department of Large Animal Diseases with Clinic, Faculty of Veterinary Medicine, Warsaw University of Life Sciences, Warsaw, Poland; Wake Forest University, School of Medicine, United States of America

## Abstract

**Background:**

Cancer spread to other organs is the main cause of death of oncological patients. Migration of cancer cells from a primary tumour is the crucial step in the complex process of metastasis, therefore blocking this process is currently the main treatment strategy. Metastasis inhibitors derived from natural products, such as, migrastatin, are very promising anticancer agents. Thus, the aim of our study was to investigate the effect of six migrastatin analogues (MGSTA-1 to 6) on migration and invasion of canine mammary adenocarcinoma cell lines isolated from primary tumours and their metastases to the lungs. Canine mammary tumours constitute a valuable tool for studying multiple aspect of human cancer.

**Results:**

Our results showed that two of six fully synthetic analogues of migrastatin: MGSTA-5 and MGSTA-6 were potent inhibitors of canine mammary cancer cells migration and invasion. These data were obtained using the wound healing test, as well as trans-well migration and invasion assays. Furthermore, the treatment of cancer cells with the most effective compound (MGSTA-6) disturbed binding between filamentous F-actin and fascin1. Confocal microscopy analyses revealed that treatment with MGSTA-6 increased the presence of unbound fascin1 and reduced co-localization of F-actin and fascin1 in canine cancer cells. Most likely, actin filaments were not cross-linked by fascin1 and did not generate the typical filopodial architecture of actin filaments in response to the activity of MGSTA-6. Thus, administration of MGSTA-6 results in decreased formation of filopodia protrusions and stress fibres in canine mammary cancer cells, causing inhibition of cancer migration and invasion.

**Conclusion:**

Two synthetic migrastatin analogues (MGSTA-5 and MGSTA-6) were shown to be promising compounds for inhibition of cancer metastasis. They may have beneficial therapeutic effects in cancer therapy in dogs, especially in combination with other anticancer drugs. However, further *in* *vivo* studies are required to verify this hypothesis.

## Introduction

**Figure 1:**
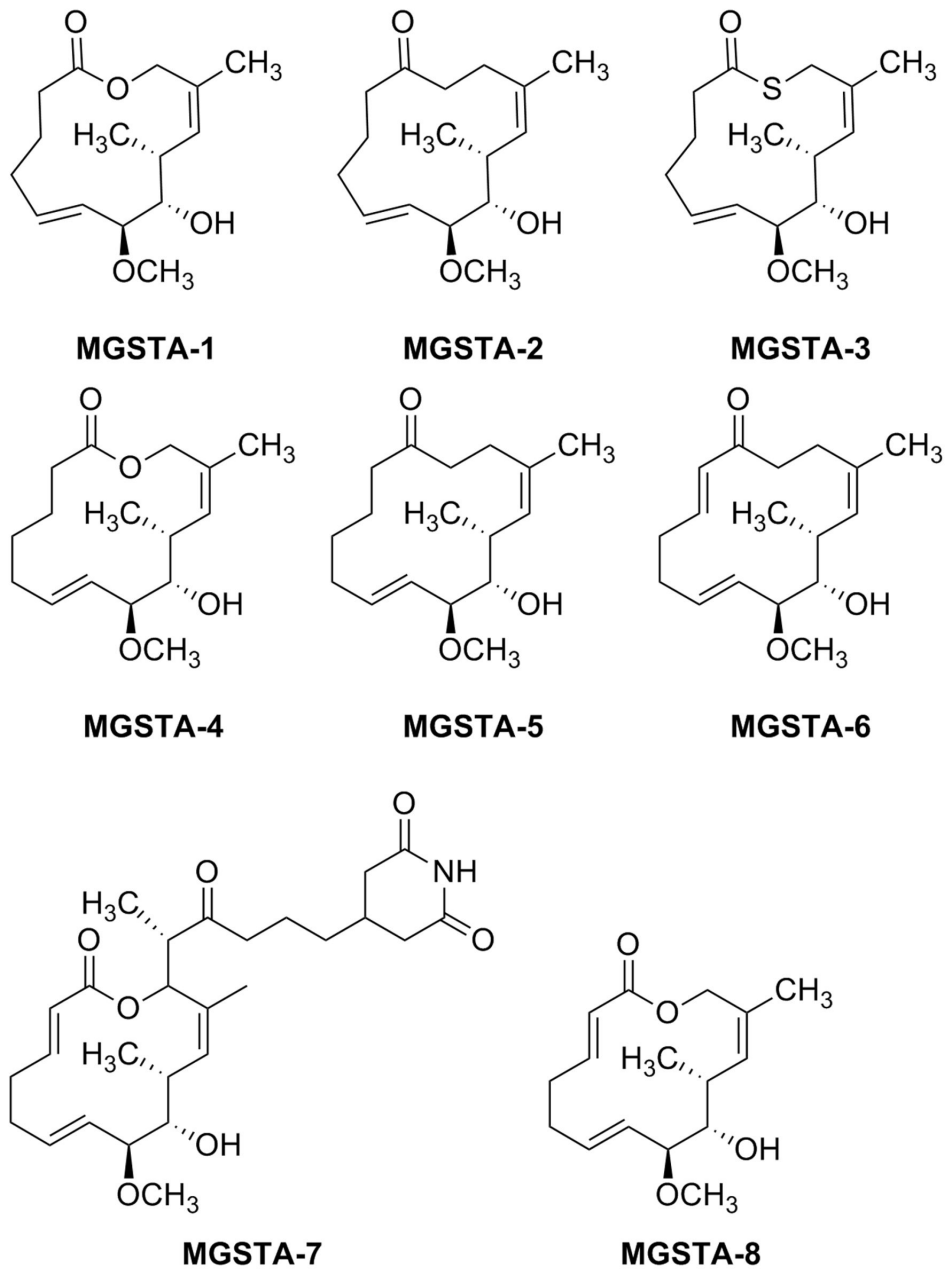
Structures of migrastatin MGST-7 and tested migrastatin analogues MGSTA-1 to 6 and MGSTA-8.

Metastasis is the cause of most cancer deaths [1,2]. Although it is extensively investigated, it still remains one of the most inscrutable aspects of the disease. Poor outcomes of current therapies, in particular poor prognosis for patients in advanced stages of solid tumours due to metastasis encourage investigators all over the world to find new drugs that could inhibit metastatic cascade. A promising agent is the natural product migrastatin: a new anti-metastatic compound of microbial origin. It is a macrolactone natural product originally isolated from *Streptomyces* sp. MK929-43F1 by Imoto et al. in 2000 [3-5] and later from *Streptomyces platensis* by Licari et al. in 2002 [6] as an inhibitor of tumour cell migration. Just few years later the first total chemical synthesis of migrastatin was reported [7,8]. Since then, several more effective analogues of migrastatin have been synthesized and described as promising anti-metastatic drugs [8-13]. There have been efforts to generate further analogues for structure activity studies [14-17]. Importantly, simpler analogues of migrastatin, such as the macrolide MGSTA-8 (Figure 1), have shown activity ~1000 fold more active than migrastatin itself in tumour cell migration assays *in vitro* [9]. Truncated analogues such as MGSTA-4 and the macroketone MGSTA-5 and MGSTA-8 inhibit metastasis of highly metastatic tumour cells in mouse models [10,12]. However, inhibition of cell migratory ability considerably depends on the structure of the compounds and some acyclic analogues show activity [11,14,15].

The molecular mechanism by which migrastatin affects cancer cell migration and invasion has not been fully discovered, although it has been proposed to target fascin1, the actin-bundling protein [18]. Migrastatin may bind to one of the actin-binding sites preventing actin cross-linking of filaments and filopodia formation [18]. Over-expression of *fascin1* in cancer cells has been previously linked with clinically aggressive character of the tumour, poor prognosis and shortened disease-free survival time [18-20].

Our previous studies have shown up-regulation of *fascin* in highly invasive canine mammary cancer cell lines [21]. Thus, we used them as a model for investigation of fascin-targeting drugs, such as migrastatin. Hence, we examined a role of six various analogues of migrastatin MGSTA-1 to 6, including the Danishefsky macroketone (MGSTA-5) and macrolactone (MGSTA-4), in canine mammary cancer cell invasion, migration and cytoskeleton rearrangement.

The canine mammary tumours are attractive and useful model to study human breast cancer because they capture the essence of human breast cancers, unlike most genetically-modified or xenograft rodent models. Firstly, dogs share the same environment as humans, thus are exposed to many of the same carcinogens. Secondly, those tumours occur in dogs naturally and spontaneously and have an identical course to that in humans. Numerous clinical similarities have been demonstrated so far, concerning the hormonal aetiology of tumours, age of onset, risk factors (e.g. obesity), clinical outcome, prognosis factors (e.g. tumour size, lymph node invasiveness and clinical stage) (for review see: [22-24];). In addition, many strong and significant similarities at the molecular level have been shown in genome-wide comparative studies. For instance, pathways related with the promotion of proliferation were up-regulated in both species, and those connected with cell development, cell matrix adhesion, and cell communication, were down-regulated [23]. Moreover, overexpression of steroid receptors, proliferation markers, epidermal growth factor, p53 suppressor gene mutations, metalloproteinases and cyclooxygenases, was found in both species, as well as great homology in the alterations of cancer-related pathways such as the phosphatidylinositol 3-kinase (PI3K)/AKT pathway, KRAS, phosphatase and tensin homolog (PTEN), Wnt-β-catenin, and the mitogen-activated protein kinase (MAPK) cascade [23,25]. Therefore, the canine mammary tumour model constitutes a valuable tool for translational medicine particularly for the evaluation and development of novel diagnostic and prognostic applications as well as therapeutic strategies that will benefit both dogs and human cancer patients [22-26].

## Methods

### Cell culture

The cell lines used for this study have been described in our previously published research [21,27]. Two adenocarcinoma cell lines were isolated from the canine mammary tumors (CMT-W1 and CMT-W2) and two cell lines were isolated from their lung metastases (CMT-W1M and CMT-W2M). The cell lines were cultivated in RPMI-1640 medium containing 10% heat-inactivated fetal bovine serum (FBS), 50 U/ml penicillin, 50 µg/ml streptomycin and 2,5 µg/ml amphotericin B (reagents obtained from Sigma Aldrich Chemical Co., USA). Cells were grown in tissue culture flasks (Nunc™, Denmark) in an atmosphere of 5% CO2 and 95% humidified air at 37°C, and were routinely subcultured every second day.

### Migrastatin analogues

The structures of tested migrastatin analogues (MGSTA-1 to 6) are presented in Figure 1.

### Synthesis of analogues of migrastatin

**Figure 2:**
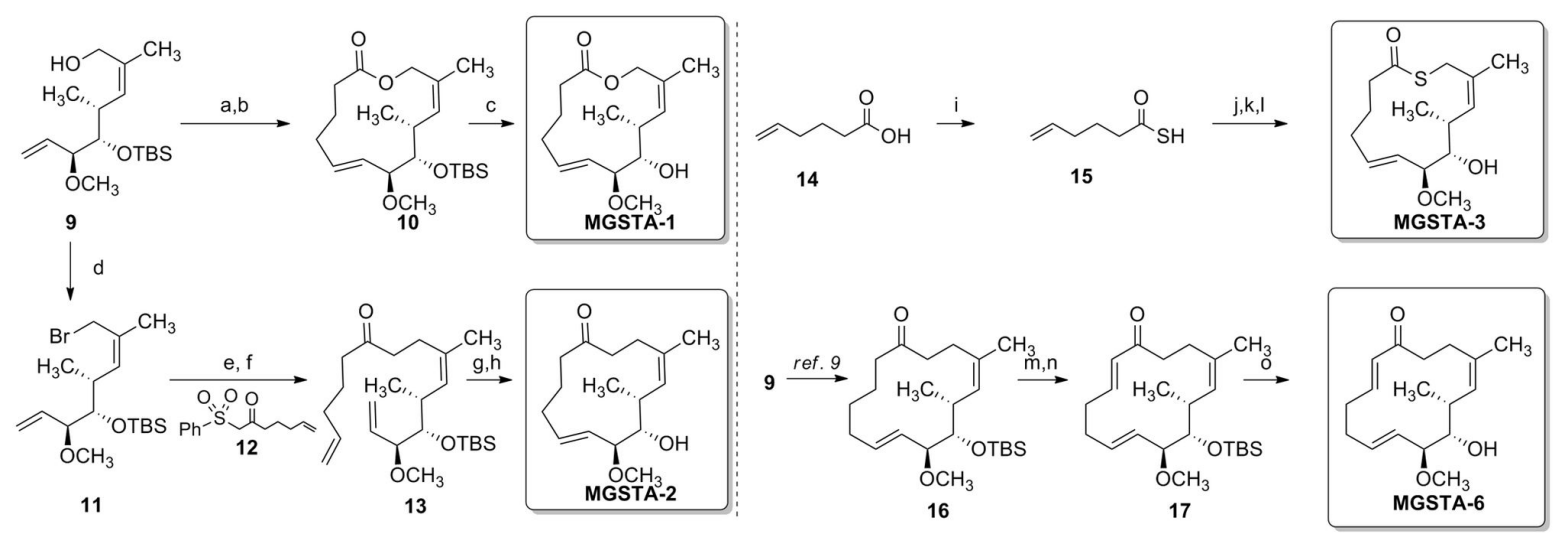
Synthesis of migrastatin-core analogues. Reagents and conditions: (a) 5-hexenoic acid, Ph _3_P, DIAD, PhCH_3_, rt, 3h, 69%; (b) Grubbs 2^nd^, PhCH_3_, reflux, 25 min, 68%; (c) HF. Py, THF, rt, 18h, 61%; (d) CBr_4_, Ph _3_P polymer-bound, CH_2_Cl_2,_ rt, 50 min; (e) **12**, DBU, PhCH_3_ then **11**, rt, 1.5h; (f) Na/Hg, MeOH, rt, 3h, 51% over three steps; (g) Grubbs 2^nd^, PhCH_3_, reflux, 15 min, 99%; (h) HF. Py, THF, rt, 38h, 84%; (i) Lawesson reagent, CH_2_Cl_2_, mw, 100 °C, 10 min; (j) **9**, Ph _3_P, DIAD, PhCH_3_, rt, 15h, 42%; (k) Grubbs 2^nd^, PhCH_3_, reflux, 15 min, 88%; (l) HF. Py, THF, rt, 18h, 85%; (m) TMSCl, LHMDS, THF, 0 °C, 2h; (n) Pd(OAc)_2_, CH_3_CN, rt, 3h, 64% over two steps; (o) HF. Py, THF, rt, 18h, 90%.

Compound MGSTA-4 and MGSTA-5 were synthesized from **9**, as reported in literature [9] while compounds MGSTA-1 to 3 and MGSTA-6 were prepared from **9** [9] as illustrated in Figure 2. Thus esterification of **9** with 5-hexenoic acid under Mitsunobu conditions followed by ring-closing metathesis (RCM) gave **10** and subsequent removal of the TBS group gave MGSTA-**1**. Treatment of **9** with carbon tetrabromide in the presence of polymer supported triphenylphosphine in dichloromethane gave the allylic bromide **11** [9]. Subsequent reaction of β-ketosulfone **12** in the presence of DBU followed by reductive removal of the sulfone group gave **13**. Ring closing metathesis and removal of the TBS group gave MGSTA-**2**. The thiolactone was prepared from **14** which was first of all converted to the thio acid **15** using Lawesson’s reagent [28]. Formation of the thiolactone under Mitsunobu conditions, RCM and TBS removal gave MGSTA-**3**. The TBS protected macroketone **16** was prepared from **9** as described previously [9]. Formation of the TMS enol ether was achieved in a regioselective manner and subsequent oxidation with Pd(OAc)_2_ gave the α,β-unsaturated macroketone **17**. Finally, removal of the TBS protecting group gave unsaturated macroketone MGSTA-**6**.

### Cell viability assay (MTT-assay)

Cell viability (metabolic activity of viable cells) was quantified by MTT assay. Cells were seeded onto 96-well plate (Nunc Inc., Denmark) at the concentration of 1 × 10^4^ cells per well. When cells reached 60-70% confluence, the medium was replaced with medium containing 10% FBS and six various migrastatin analogues (MGSTA-1 to 6) at the concentrations of: 1, 10 or 100 µM. The cultures were incubated for 24 hours. Then, cells were incubated with 0.5 mg/ml tetrazolium salt MTT diluted in phenol red-free RPMI 1640 medium (Sigma Aldrich) for 4 hrs at 37°C. To complete solubilization of the formazan crystals, 100 µl of DMSO (Dimethyl sulfoxide, Sigma Aldrich) were added to each well. Cell viability was quantified by measuring photometric absorbance at 570 nm in a multi-well plate reader (Infinite 200 PRO Tecan™, TECAN, Switzerland). All the samples were examined in triplicate, each experiment was conducted three times (n= 9).

### Cell culture on a reconstituted basement membrane (Matrigel)

Cancer cells were treated with trypsin and resuspended in culture medium. Each well of the Lab-Tek 8-chamber culture slides (Nunc Inc., USA) was coated with 25 µl of growth factor reduced Matrigel (BD Biosciences) and the slides were left to solidify for 30 min. at 37°C. The cells were then plated at a concentration of 10^4^ cells/mL in migrastatin analogue-containing medium or medium with addition of DMSO (drug vehicle). The growth of cells on Matrigel was observed everyday under phase-contrast microscope (IX 70 Olympus Optical Co., Germany). The experiment was conducted three times (n= 3).

### *In vitro* wound healing assay (scratching test)

The scratch assay was conducted to assess the influence of migrastatin analogues on canine mammary carcinoma cell motility. The cells were seeded in a high density at 600 mm Petri dishes (Corning-Costar, Cambridge, USA). After 24 hrs, the medium was removed and replaced with medium containing 10% FBS and one of six migrastatin analogues (MGSTA-1 to 6) or DMSO (control). The monolayer was wounded by scratching the surface: as uniformly and straight as possible with a pipette tip (1000 µl) at least three times. The images of cells invading the scratch were captured at indicated time points (0, 4, 6, 8 and 24 hrs) using phase contrast microscopy (IX 70 Olympus Optical Co., Germany). The pictures were analyzed using a computer-assisted image analyzer (Olympus Microimage™ Image Analysis, software version 4.0 for Windows, USA). The migration rate was expressed as percentage of scratch closure and was calculated as follows: % of scratch closure= a-b/a, where (a) is a distance between edges of the wound, and (b) is the distance which remained cell-free during cell migration to close the wound [29].

The values are presented as means of three independent wound fields from three independent experiments (n= 9).

### Trans-well migration assay

Boyden chamber cell migration assay was performed using the BD Falcon™ FluoroBlock™ 24-Multiwell Insert Plates (8 micron pore size) (BD Biosciences, USA). Firstly, cells (1×10^5^) were suspended in FBS free medium and added to the apical chambers of an insert plates (500 µl). Then 750 µl of chemoattractant (10% FBS) was added to the basal chambers. Migrastatin analogues (MGSTA-5 or MGSTA-6 at 100 µM) or DMSO (as a control) were added to the medium in both chambers. For dose-dependent studies MGSTA-6 was used in the range of concentration from 0.1 to 100 µM. Migration assays were carried out for 18-20 hours at standard culture conditions. Then, medium was carefully removed from apical chamber and the insert system was transferred into a second 24-well plate containing 500 µl of 2.5 µg/ml Calcein AM in Hanks’ Balanced Salt solution (HBSS). Plates were incubated for one hour at standard culture conditions. Then, the fluorescence of migrated cells was measured at excitation wavelength 485 nm and emission wavelength 530 nm using florescent plate reader with bottom reading capabilities Infinite 200 PRO Tecan™ (TECAN, Switzerland). All samples were assayed in triplicate, and each experiment was conducted three times (n= 9). For each experiment two negative controls were used: (1) cells grown as given above without adding chemoattractant to the medium, and (2) medium added as described without cells. In order to determine fluorescence of cells that migrated through the membrane, plates were analysed using fluorescence microscope (Olympus BX60, magnification x4).

### Trans-well invasion assay

The cell invasion assay was performed using the BD BioCoat™ 24-Multiwell Invasion System pre-coated with BD Matrigel™ Matrix (BD Biosciences, USA). The insert plates were firstly prepared by rehydrating the BD Matrigel™ Matrix layer with warm PBS for two hours at 37°C. The rehydration solution was then carefully removed, and 500 µl of cell suspension was added to the apical chambers (2.5 × 10^5^ cells). Cell suspension was prepared by trypsinizing cell monolayers (80% confluent) and resuspending the cells in FBS free medium. Then 750 µl of chemoattractant (10% FBS) was added to the basal chambers. For the experiment, MGSTA-6 or DMSO (as a control) were added to the medium in both chambers. Invasion assay plates were incubated for 22-24 hours at standard culture conditions. Then, medium was carefully removed from apical chamber and insert system was stained with Calcein AM in HBSS in the same way as described in migration assay. Then, the fluorescence of invaded cells was measured using the florescent plate reader Infinite 200 PRO Tecan™ (Ext.485 nm/Ems. 530 nm) (TECAN, Switzerland). All samples were assayed in triplicate, each experiment was carried out three times (n= 9). For each experiment the negative control constituted medium where chemoattractant was not added. In order to determine fluorescence of cells that invaded through the membrane coated by Matrigel the plates were analysed using fluorescence microscope (Olympus BX60, magnification x4).

### Confocal microscopy

The canine mammary cancer cells were grown on Lab-Tek culture slides coated with Matrigel for 24 hrs in migrastatin containing medium (MGSTA-6 at the concentration of 100 µM). Then, cells were washed twice in warm PBS and fixed with 3.7% paraformaldehyde for 20 min at room temperature (RT). Next, the cells were permeabilized with 0.5% Triton X-100 diluted in PBS (10 min at RT), washed in PBS three times and incubated with primary rabbit anti-phospho-FSCN1(Ser39) antibody at 1:100 dilution in PBS (Bioss, USA). After overnight incubation with primary antibodies at 4°C cells were washed three times in PBS and incubated with secondary Alexa Fluor 568 goat anti-rabbit antibodies diluted 1:500 in PBS (Santa Cruz Biotechnology Inc., USA) and co-stained for F-actin with FITC–X phalloidin diluted 1:100 in PBS (Invitrogen, USA) for an hour in darkness at RT. The coverslips were then mounted on microscope slides using SlowFade®Gold mounting medium (Life Technologies, Invitrogen). Cells were visualized using the confocal laser scanning microscope FV-500 system (Olympus Optical Co, Hamburg, Germany). The combination of excitation/emission were: Argon 488 nm laser with 505-525 nm filter for FITC and HeNe 543 nm laser with 610 nm filter for Alexa Fluor 568 staining. The pictures were gathered separately for each fluorescence channel. The cells were examined using the Fluoview program (Olympus Optical Co, Germany). To quantify the confocal results, we have analyzed the intensity of red fluorescence related to fascin1 expression and colorimetric intensity of spots that show co-localization of fascin1 and F-actin at merge images, using computer-assisted image analyser (Olympus Microimage™ Image Analysis, software version 5.0 for windows, USA). Five to ten pictures in each slide were analysed. The antigen spot colour intensity was expressed as mean pixel integrated optical density (IOD).

### Statistical analysis

The statistical analysis was conducted using GraphPad Prism version 5.00 software (GraphPad Software, USA). The one-way ANOVA and Tukey HSD (Honestly Significant Difference) post-hoc test, Dunnett’s test and *t*-test were applied as well as regression analysis. The p-value < 0.05 was regarded as significant whereas p-value < 0.01 and < 0.001 as highly significant. The data was expressed as means +/- S.D. unless otherwise stated. The *in vitro* wound healing assay was analysed using two-way RM ANOVA and Bonferroni post-hoc test.

## Results and Discussion

### The effect of six analogues of migrastatin on cancer cell viability

Firstly, we investigated if the examined analogues of migrastatin affected cell viability. Four of these chemical compounds (MGSTA-1, MGSTA-3, MGSTA-5, and MGSTA-6) did not change the viability of canine cancer cells at any used concentration (Figure S1). However, the viability of the CMT-W1 and CMT-W2 cells significantly decreased (by approximately 29% and 32%, respectively) after treatment with MGSTA-4 at the concentration of 100 µM (p < 0.01) (Figure S1A, B). The viability of the CMT-W1 cells also decreased after incubation with MGSTA-2 by approximately 26% (Figure S1A). The decrease of cell viability after treatment with 100 µM of MGSTA-4 and MGSTA-2 might have been related with their cytotoxicity, therefore, in the following experiments, these compounds were used at the concentration of 10 µM to ensure that the observed effect of these compounds on migration and invasion was not resulting from inhibition of cell proliferation or induction cell death.

Conversely, we also observed, that lower concentrations of other analogues of migrastatin caused a slight increase in cancer cell viability (e.g. MGSTA-5 or MGSTA-6 in the CMT-W1 cell line; MGSTA-1, MGSTA-2 in the CMT-W2 cell line and MGSTA-3 in the CMT-W1M and CMT-W2M cell lines) (Figure S1). Thus, to avoid possible cancer cell growth promotion by treated compounds, in following experiments we used the highest possible concentration (100 µM) that did not affect the cell viability (nor decreasing neither increasing its viability). Furthermore, previous studies conducted on human A549 lung cancer cell line demonstrated that lower concentrations of migrastatin analogues (ME - migrastatin core ether) did not affect cell migration [13]. Additionally, a study by Shen and co-workers [11], in which eighteen different semi-synthetically produced migrastatin congeners were investigated, revealed that only two compounds displayed cell migration inhibition IC_50_ values below 100 µM in the wound healing assay.

### Inhibition of canine mammary cancer cells migration by analogues of migrastatin evaluated by wound healing assay *in vitro*

**Figure 3:**
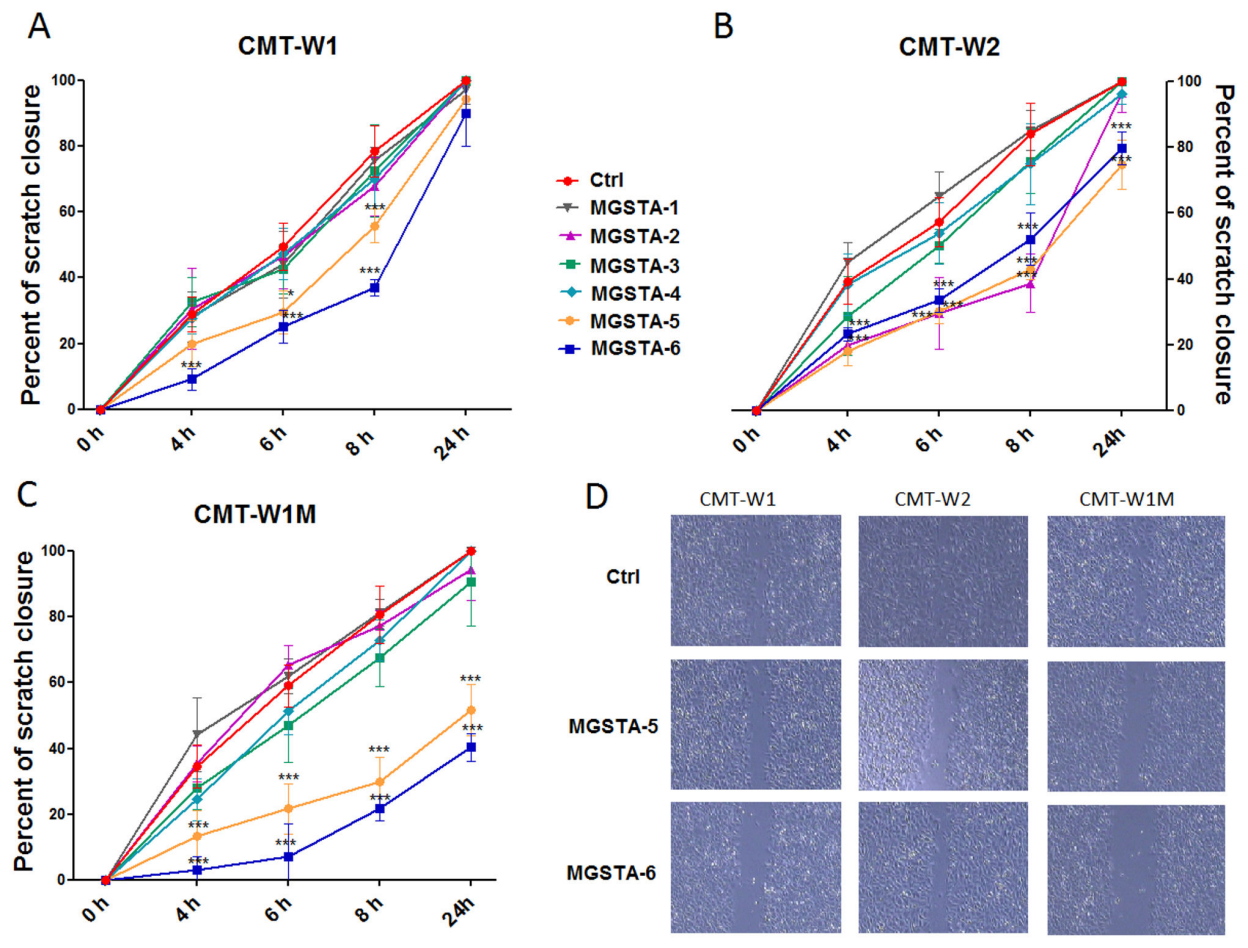
Wound healing assay of canine mammary carcinoma cells in control conditions and after treatment with six migrastatin analogues (MGSTA-1 to 6). Quantification of migration of CMT-W1 (A), CMT-W2 (B) CMT-W1M (C) cell lines are presented as percentages of scratch closure and were calculated as follows: % of scratch closure = a-b/a, where (a) is a distance between edges of the wound, and (b) is the distance which remained cell-free during cell migration to close the scratch. Photographs of cells invading the scratch were taken at the starting point and after 4, 6, 8, and 24 hrs using phase contrast microscopy (IX 70 Olympus Optical Co., Germany). Administration of MGSTA-5 and MGSTA-6 (100 µM) decreased cells motility in CMT-W1, CMT-W2 and CMT-W1M cell lines. The MGSTA-2 treatment decreased the cells motility only in the CMT-W2 cell line. Other migrastatin analogues do not affect migration ability of examined cell lines. (D) Representative images of scratch closure in control conditions and after MGSTA-5 and MGSTA-6 treatment (100 µM) at the 8^th^ hr after scratching in CMT-W1, CMT-W2 and CMT-W1M cell lines; images were taken using lens with 4x magnification and analysed using a computer-assisted image analyser (Olympus Microimage™ Image Analysis, software version 5.0 for Windows, USA). The experiment was performed three times. Two-way ANOVA and Bonferroni post-hoc tests were applied. p < 0.05 was marked as * and p < 0.001 was marked as ***.

The wound healing assay was performed in order to characterize the effect of analogues of migrastatin on the migration of canine cancer cells. As shown in the Figure 3 two of six examined compounds (MGSTA-5 and MGSTA-6) caused strong inhibitory effect on cancer cell migration in the CMT-W1, CMT-W1M and CMT-W2 cell lines. In addition, also MGSTA-2 was quite effective in the CMT-W2 cell line at the time points 4, 6 and 8 hours after scratching. While in control condition cells closed 39.2±6.8%, 57±7.5% and 84±9.4% of the wound (after 4, 6 and 8 hrs, respectively), after MGSTA-2 administration cells closed only 19.9±2.1%, 29.6±9.9% and 38.9±8.8% of the wound, respectively. However, after 24 hours the wound was completely closed as in control condition (Figure 3B). Cell migration was not affected by any of the examined migrastatin analogues in the CMT-W2M cell line (data not shown).

The MGSTA-5 given at the concentration of 100 µM inhibited migration of CMT-W1, CMT-W1M and CMT-W2 cells in response to the scratch wound. The CMT-W1 cells treated with MGSTA-5 closed only 29±6.5% and 56±5.2% of the wound (after 6 and 8 hrs, respectively), whilst in control conditions, 50±7.2% and 78±7.8% of the wound was closed 6 and 8 hrs after scratching, respectively (Figure 3A). Similarly, CMT-W2 cells incubated with MGSTA-5(100 µM) closed only 30±3.8% and 43±3% of the wound 6 and 8 hrs after scratching (respectively), whereas in control conditions they closed 57±7.5% and 84±9.4% of the wound at that time (6 and 8 hrs respectively) (Figure 3B). Similarly, also CMT-W1M cells incubated with this compound closed 21±7.7% and 30±7.4% of the wound 6 and 8 hrs after scratching, respectively, whereas control cells closed 59±6.3% and 80±8.7% of the wound at the same time points (Figure 3C). Moreover, the CMT-W2 and CMT-W1M cell lines treated with MGSTA-5 did not close the wound even after 24 hrs of scratching (74±7.3% and 52±7.8% closure, respectively). In control conditions, the wound was completely closed (Figure 3B, C).

The MGSTA-6 also proved to be a very effective inhibitor of migration. The CMT-W1 cell line treated with this agent closed only 25±5.1% and 37±2.8% of the wound 6 and 8 hrs post-scratching (Figure 3C). Treatment of CMT-W2 cells with MGSTA-6 decreased also their migration ability. It was highly significant 8 and 24 hrs after scratching. In control conditions the wound was almost completely closed at that time (86-100%), whereas cells treated with MGSTA-6 closed only 52±8.1% and 78±5.2% of the wound, respectively (Figure 3B). The MGSTA-6 caused a similar effect in CMT-W1M cell line, which was manifested by almost complete block of migration (9±6.8% and 21±3.7% of wound closure 6 and 8 hrs after scratching, respectively). After 24 hrs only 41±4.2% of the wound was closed (Figure 3C).

Danishefsky and co-workers obtained similar results [13]. The authors investigated anti-metastatic properties of migrastatin core ether (ME) and carboxymethyl- migrastatin ether (CME). Both compounds given at the concentration of 100 µM almost completely blocked migration of human non-small-cell lung carcinoma 12 hrs after scratch. The authors based on wound healing assay determined the half maximal inhibitory of migration concentration (IC_50_ value), which was lower than obtained in our research with using trans-well migration assay (discussion below). The most effective inhibition of migration in three canine cancer cell lines, evaluated by wound healing assay was achieved with MGSTA-5 and MGSTA-6 at concentration of 100 µM, and these two were used to further characterization. Although the MGSTA-2 inhibited the migration ability of CMT-W2 cells it was inefficient in other cell lines. In turn, the MGSTA-4 (Danishefsky 'macrolactone') at high concentration of 100 µM significantly inhibited cellular proliferation of two cell lines (CMT-W1 and CMT-W2) (Figure S1A, B), but at lower concentration (10 µM) had no effect on cell migration (Figure 3). These discrepancies may be due to different characteristic of canine cell lines.

### Inhibition of migration and invasion of canine mammary cancer cells by two the most effective analogues of migrastatin (MGSTA-5 and MGSTA-6) evaluated by Boyden chambers assay

To confirm anti-metastatic activity of two compounds that were the most effective in wound healing assay, we performed the migration and invasion assays in Boyden chambers.

**Figure 4:**
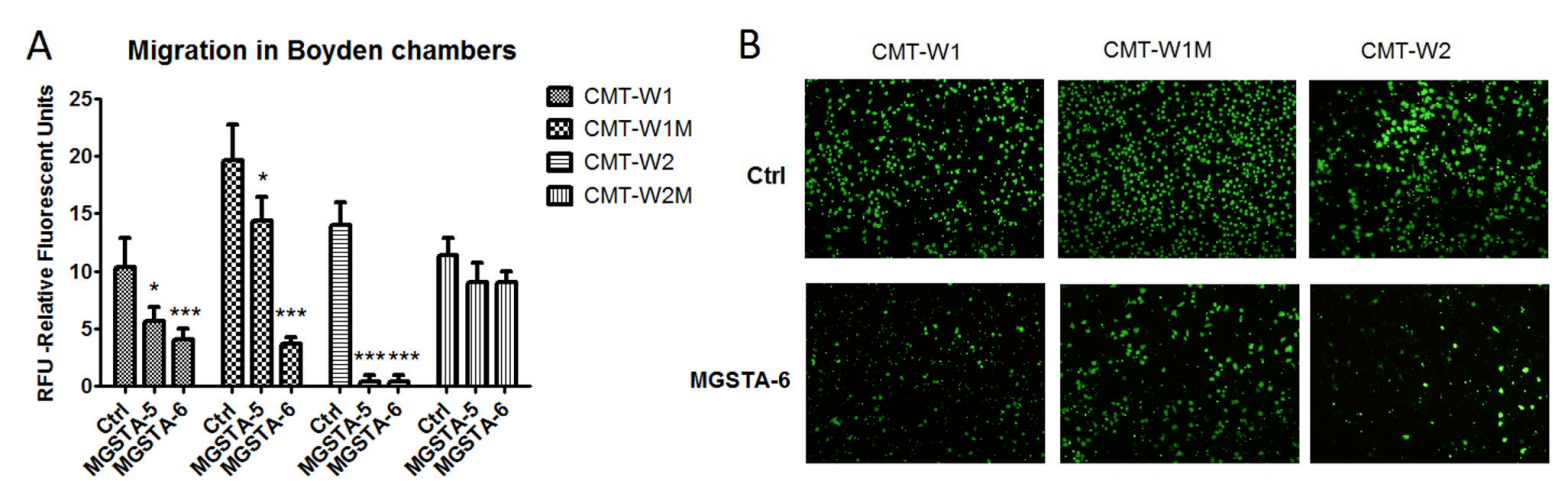
The effect of MGSTA-5 and MGSTA-6 on migration of canine mammary carcinoma cells evaluated by trans-well migration assay (A) Quantification of migration of examined cell lines (CMT-W1, CMT-W1M, CMT-W2, CMT-W2M) is presented as Relative Florescent Units (RFU) obtained using florescent plate reader Infinite 200 PRO Tecan™ (Ex. 485, Em. 530). The treatment of cancer cells with MGSTA-5 and MGSTA-6 for 20 hrs at the concentration of 100 µM decreased the migration through membrane of CMT-W1, and CMT-W1M cells and almost completely abolished migration of CMT-W2 cells. In CMT-W2M cell line those compounds were ineffective. The experiment was performed three times. The statistical analysis was done using Prism version 5.00 software (GraphPad Software, USA). One-way ANOVA followed by Tukey HSD post-hoc test and unpaired t-test were applied. p < 0.05 was marked as *, p < 0.001 was marked as ***. (B) Micrographs of the migrated CMT-W1, CMT-W1M and CMT-W2 cells taken with Olympus microscopy BX60 at x4 magnification.

Cancer cell migration was significantly decreased after 24 hours incubation with 100 µM of MGSTA-5 and MGSTA-6 in CMT-W1 and CMT-W1M cell lines. The fluorescence intensity related to the migrating cells in control conditions was 10.3±2.5, and 19.7±3.1 in CMT-W1 and CMT-W1M cell line, respectively, whereas, treatment with macroketone MGSTA-5 decreased the relative fluorescent units (RFU) to 5.6±1.2 and 14.2±2,1 in CMT-W1 and CMT-W1M cell line, respectively (Figure 3A). A similar effect was obtained in cells treated with MGSTA-6. RFU decreased to 4.2±1.2 and 3.8±1 in CMT-W1 and CMT-W1M cell line, respectively (Figure 4A). Migration of CMT-W2 cells was almost completely inhibited by these compounds. However, no effect on migration of the CMT-W2M cells was noted, although a slight decrease was observed (Figure 4A).

**Figure 5:**
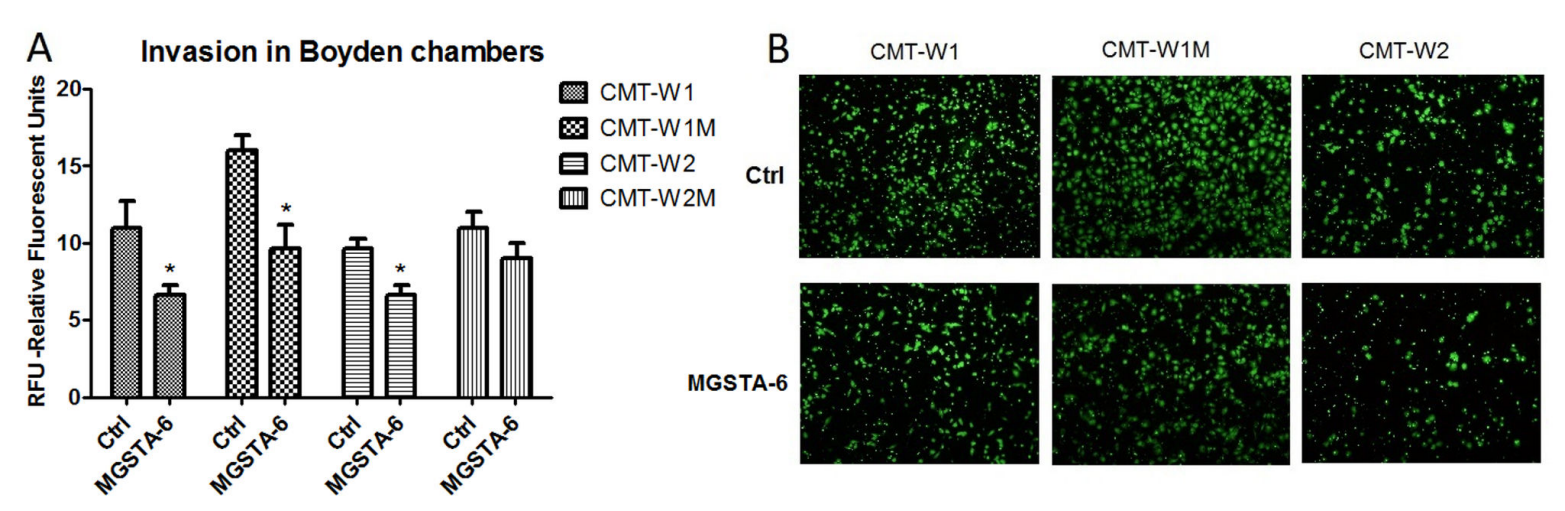
The effect of MGSTA-6 on invasion of canine mammary carcinoma cells evaluated by trans-well invasion assay. (A) Quantification of invasiveness of examined cell lines (CMT-W1, CMT-W1M, CMT-W2, CMT-W2M) is presented as Relative Florescent Units (RFU) obtained using fluorescent plate reader Infinite 200 PRO Tecan™ (Ex. 485, Em.530). The treatment of cancer cells with MGSTA-6 for 24 hrs at the concentration of 100 µM decreased the invasiveness through membrane pre-coated by Matrigel™ Matrix, of CMT-W1M, CMT-W2, CMT-W1M cells. In CMT-W2M cell line MGSTA-6 was ineffective. The experiment was performed three times. The statistical analysis was done using Prism version 5.00 software (GraphPad Software, USA). One-way ANOVA followed by Tukey HSD post-hoc test and unpaired t-test were applied. p < 0.05 was marked as *. (B) Fluorescence microscopic analysis of the invaded through Matrigel layer CMT-W1, CMT-W1M and CMT-W2 cancer cells using Olympus microscopy BX60 at 4x magnification.

The cancer cell invasion assay was performed only with the most potent analogue of migrastatin (MGSTA-6). The invasiveness of CMT-W1, CMT-W1M and CMT-W2 canine cancer cell lines was significantly decreased by this compound administered at the concentration of 100 µM for 24 hours. The fluorescence intensity related to the invaded CMT-W1, CMT-W1M and CMT-W2 cells in control conditions was 11±1.7, 9.7±0.6, and 11.4±1.5, respectively (Figure 5A). Administration of MGSTA-6 decreased the invasiveness of cancer cells. The florescence intensity related to the invaded CMT-W1, CMT-W1M and CMT-W2 cells was 6.7±0.6, 6.6±0.6, 7.3±0.7, respectively (p < 0.005). The invasion of CMT-W2M cell was not affected by MGSTA-6. The microscopic analysis conducted using fluorescence microscopy confirmed the obtained results (Figures 4B and 5B).

For further characterization of MGSTA-6 dose-response studies have been carried out to determine the migration half maximal inhibitory concentration (IC_50_ value) using trans-well migration assay. As shown in Figure S2 the IC_50_ of MGSTA-6 on serum-induced migrations of CMT-W1, CMT-W1M and CMT-W2 canine mammary cancer cells was 66.11 µM, 54.89 µM and 51.10 µM, respectively. These IC_50_ values were higher than previously reported for other migrastatin analogues in human and mouse cancer cell lines [10-13]. For instance, the studies of Danishefsky on three different lung cancer cell lines with ME and CME at the concentration ranging from 10^-3^ to 10^-8^ M showed lower IC_50_ values comprising concentrations from 1.5 to 8.2 µM and from 0.5 to 5 µM for ME and CME, respectively [13]. Their previous studies showed that other analogues of migrastatin (macroketone – MGSTA-5 - and macrolactone – MGSTA-4 - core structures) had even lower IC_50_ values (100 nM and 255 nM, respectively) in mouse mammary cancer cell line 4T1 and only slightly higher IC_50_ values (350 nM and 2.7 µM, respectively) in human breast cancer cell line [10]. Interestingly, migration of normal mammary gland epithelial cells as well as mouse fibroblasts and leukocytes was hardly affected by even very high concentrations of migrastatin (IC_50_ >200 µM), which indicated that these compounds were selective for metastatic tumour cells [10]. Similar effect was reported by Shan and co-workers [11]. The authors demonstrated, that two migrastatin analogues produced semi-synthetically from biosynthesized 12-membered macrolides were very promising anti-metastatic compounds with cell migration inhibition IC_50_ values of 1.8 µM and 70 nM [11]. In this study cytotoxic IC_50_ values of the compounds were also relatively low (5.2 µM and 1.0 µM, respectively), although these cytotoxic IC_50_ concentrations were above those found for cell migration inhibition [11]. In our research the migratory IC_50_ values were much lower than used (non-toxic) what indicated that anti-migratory effect was not caused by inhibition of cancer cells proliferation but changed their abilities to spread.

### Inhibition of Branch Formation by MGSTA-6 in Canine Mammary Cancer Cells Grown on Matrigel Matrix

**Figure 6:**
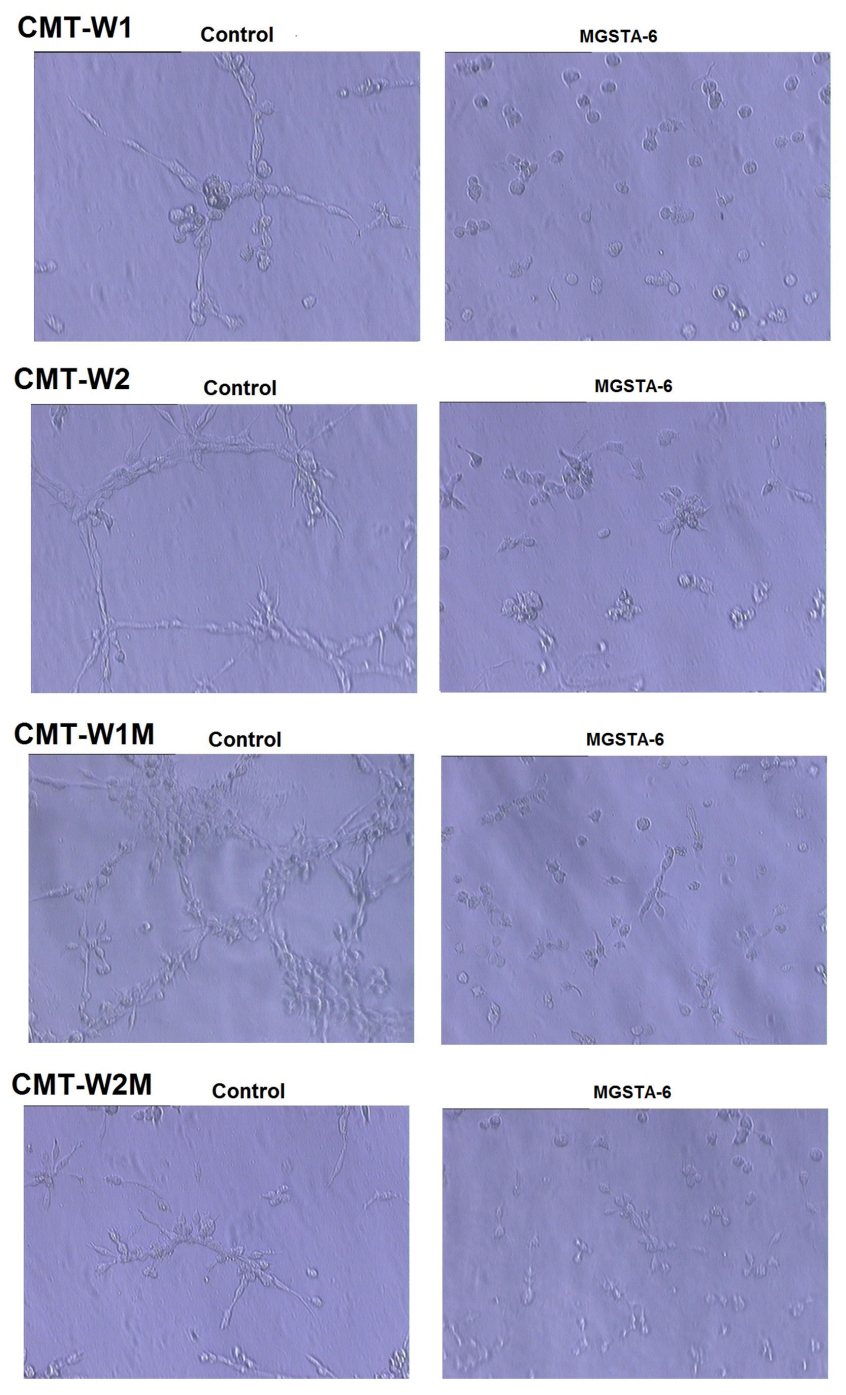
Growth characteristics of CMT-W1, CMT-W2, CMT-W1M and CMT-W2M cell lines cultured on the Matrigel Matrix. Cell were incubated with MGSTA-6 or grown in control conditions for 24 hrs on 8-chamber culture slides coated with Matrigel™ Matrix (BD Biosciences, USA). In control condition cells presented branched shape, with numerous filopodia, typical for those invasive cells growing on Matrigel. The CMT-W1, CMT-W1M and CMT-W2 cells formed linear bindings, which created polygonal structures. The CMT-W2M cells formed lower amount of branches and did not created such structures. Administration of MGSTA-6 caused very potent inhibition of branches formation in CMT-W1, CMT-W1M and CMT-W2 cancer cell lines and decreased the amount of branches in CMT-W2M cell line. The round bands, packets of cells were observed in all fields of view after MGSTA-6 treatment. The micrographs were obtained with phase-contrast microscope at magnification of x4 (IX 70 Olympus Optical Co., Germany).

In order to determine the mechanism of action of the most potent migrastatin analogue MGST-6 in canine mammary cancer cells we examined the phenotype of the those cells cultured on reconstituted basement membrane (Matrigel^TM^ Matrix) in the presence of MGSTA-6 at 100 µM (the most effective concentration, without cytotoxicity) (Figure 6). The invasive phenotype of all the examined cell lines has been indicated previously [30,31]. After 24hrs of culturing on Matrigel in control conditions all examined cell lines presented the typical branching formation. In addition, the CMT-W1, CMT-W1M and CMT-W2 formed linear bindings, which created polygonal structures. The amount of branches was slightly lower in CMT-W2M cell line compared to other cell lines and CMT-W2M cells did not form any polygonal structures. Interestingly, in all examined cell lines treated with MGST-6, the formation of branches was almost completely inhibited, which was observed in all fields of view (Figure 6). Cells grew as round colonies or packets composed of a few cells. Therefore we decided to assess changes in actin machinery, which are responsible for filopodia formation and cell motility.

### Interference with fascin1 dependent actin cross-linking in canine mammary cancer cells by analogue of migrastatin 6 (MGSTA-6)

**Figure 7:**
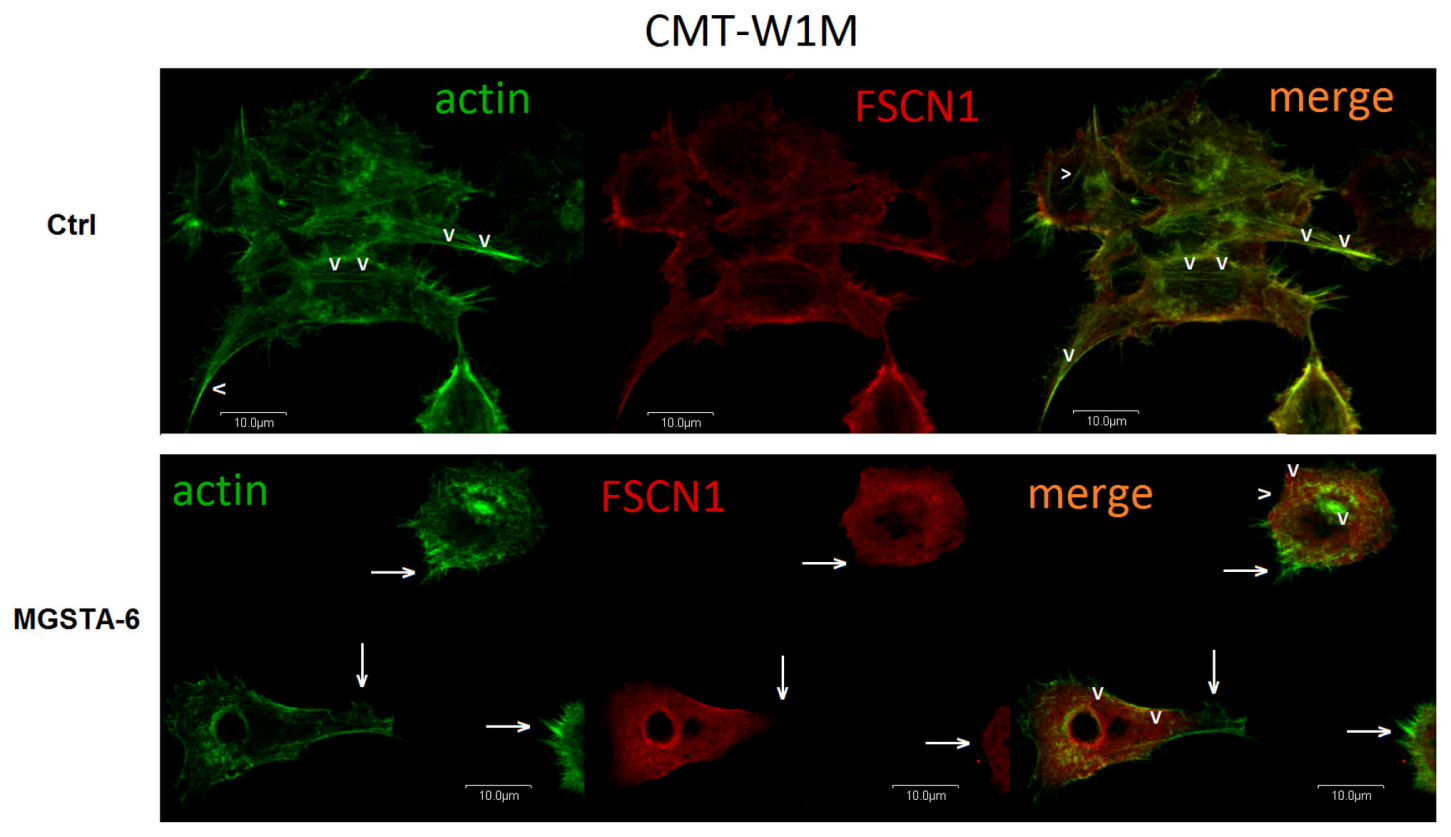
Representative confocal microscopy images of cytoskeletal protein F-actin and fascin 1 in CMT-W1M canine carcinoma cell line. The images demonstrated actin fibril (green) a fascin1 (red) localization in control conditions (upper row) and after MGSTA-6 treatment (lower row). In control condition multiple filopodia protrusion was observed as well as stress fibers (arrowheads in upper row). Moreover expression of fascin1 strongly co-localized with F-actin (merge image in upper row). In contrast, administration of MGSTA-6 caused potent inhibition of filopodia and stress fibers formation. Furthermore, the lack of expression of fascin1 in branching structures and filopodia protrusion was also shown after MGST-6 treatment (arrows in lower row). In addition, more free-fascin1 protein (not associated with F-actin) was observed in the central area of cells (arrowheads in lower row). Cells were visualized using the confocal laser scanning microscope FV-500 system at the magnification of x60, zoom 2.0 (Olympus Optical Co, Hamburg, Germany).

Fascin is one of the factors that regulate the actin cytoskeleton and it is implicated in filopodia formation. It is an actin-bundling protein, crucial for creation and maintenance of straight and tight F-actin bundles of filopodia [32-34]. Recently it has been demonstrated that migrastatin analogues directly interact with fascin1 and interfere with its binding to the actin filaments [18]. In order to investigate whether the unsaturated macroketone MGSTA-6 inhibit fascin1 activity in canine adenocarcionoma cells we used confocal microscopy. As illustrated in Figure 6 fascin1 strongly co-localized with F-actin in canine CMT-W1M control cells, whereas after incubation with MGSTA-6 more free-fascin1 protein (not associated with F-actin) was observed in the cells (Figure 7 arrowhead in bottom row). Furthermore, invasive cancer cells in control conditions had numerous filopodia, whereas, administration of MGSTA-6 caused decrease in the number of filopodial protrusions in CMT-W1M cells (Figure 7). The same effect was seen in CMT-W1 and CMT-W2 cell lines (Figure S3A, B). The CMT-W2M cells even in control conditions do not have many filopodial protrusions and stress fibers. Although in those cells after incubation with MGSTA-6 the fascin was localized at the cell periphery (Figure S3C). To quantify the confocal results, we have analysed the colorimetric intensity of spots that show co-localization of fascin1 and F-actin at merge images, using computer-assisted image analyser. The results indicated that administration of MGSTA-6 caused reduction of co-localization of these two proteins in CMT-W1, CMT-W1M and CMT-W2 cells. However, in CMT-W2M the co-localization of both protein was not significantly affected (Figure S3D).

Confocal microscopy analysis of CMT-W1M cells also revealed that after administration of the unsaturated macroketone MGSTA-6 only the F-actin protein could be detected in branching structures and filopodia protrusions, and this cytoskeletal protein did not co-localize with fascin1 (Figure 7 arrows). The obtained results are significant, considering the fact that so far fascin1 is the only known actin-bundling protein localized along the entire length of filopodia and its depletion by small interfering RNA (siRNA) caused a significant decrease in the number of filopodia [32,34]. Thus, it indicated that the unsaturated macroketone MGSTA-6 inhibits the formation and elongation of filopodia by interfering with fascin1-dependent cross-linking of actin filaments, which leads to inhibition of cell migration and invasion*.*

In addition, treatment of cells with this migrastatin analogue caused the inhibition of stress fibers formation in CMT-W1M cells (Figure 7 arrowhead in upper row) as well as in CMT-W1 and CMT-W2 cells (Figure S3A, B). Stress fibers are essential in the migration process because they provide the contractile force that causes the movement of the cell. These structures consist only of actin filaments and myosin II bundles [33]. Their loss may indicate the existence other targets of migrastatin analogues action, different from fascin1. Especially when we consider the fact that it is likely that other, currently unidentified, proteins and factors have an important role in filopodia formation [33]. The implication that different migrastatin analogues might target proteins other than fascin was firstly suggested by Danishefsky and co-workers in 2011 [13]. These authors suggested that primary protein target of migrastatin analogues (at least towards the ME and CME) might be located on the cell surface rather than intracellularly, like fascin1 [13]. However, this issue needs further investigation.

In order to determine why CMT-W2M cell line was migrastatin insensitive we have analysed fascin1 expression in all the cell lines in control conditions using the images obtained with confocal microscopy. The analysis showed that the level of phospho–fascin1 protein (phospho-FSCN1(Ser39)) was lower in CMT-W2M cells compared with other cell lines (Figure S4). The highest expression of phospho-fascin1 was found in CMT-W1M cells (p < 0.05). The lower level of fascin1 expression in CMT-W2M cells might be responsible for the ineffectiveness of MGSTA-6. However, other unknown mechanism cannot be ruled out.

## Conclusion

We showed that from six synthetic analogues of migrastatin only two (the macroketone MGSTA-5 and its unsaturated macroketone analogue MGSTA-6 (UMK)) significantly inhibited migration of three canine adenocarcioma cell lines, assessed by wound healing test and trans-well assay. Furthermore, the most effective compound (MGSTA-6) also significantly decreased the invasion of three examined canine cancer cell lines, evaluated using Matrigel coated Boyden chambers. Unsaturated macroketone MGSTA-6 has also antibranching activity and disrupts the mechanism of filopodia assembly preventing the fascin1-dependent cross-linking of actin filaments, which was demonstrated in confocal microscopy analysis of cells cultured on Matrigel. Taken together, these data indicate that migrastatin analogue 6 (MGSTA-6) impairs canine cancer cells spreading and in the future it might be a promising candidate for blocking tumour metastasis in anticancer therapy in dogs, especially in combination with compounds that would inhibit growth and development of neoplasm. However, further *in vivo* studies using animal models are required to verify this hypothesis. Structurally this compound differs from the macroketone studied *in vivo*, thus far by the presence of an additional double bond in the macrocycle. This α,β- unsaturated carbonyl group is also present in the migrastatin structure itself and could be an important structural feature the development of more potent analogues of migrastatin. The mechanism of action of migrastatin is still unclear with, as previously mentioned, fascin being implicated [18]. While the target may be fascin, or an entirely different protein, it may be the case that the presence of the α,β - unsaturated carbonyl group facilitates a Michael reaction with thiols of a cysteine group of a target protein, leading to an increase the potency of the compound compared to structures that might only have non-covalent interactions. Such Michael reactions and the formation of covalent complexes are known to occur between a subset of kinases and some natural products, for example 35. Alternatively the presence of *sp* ^2^ carbon as opposed to *sp* ^3^ carbon atoms may lead to change in the conformation of the macrocyclic ring leading to a more optimal conformation for interaction of the macrocycle with its target protein.

## Supporting Information

Figure S1 **Influence of analogues of migrastatin on viability of canine mammary cancer cells.** The canine mammary cancer cells viability was determined by MTT assay in control conditions (ctrl) and in the presence of six migrastatin analogues given at concentrations of 1, 10, or 100 µM for 24 h. Absorbance measured at 570 nm was converted to percentages (±S.D.). The experiment was conducted in n=9 repetitions. No significant differences were observed after incubation with MGSTA-1, 3 and 5-6 in CMT-W1 (A), CMT-W2 (B), CMT-W1M (C) and CMT-W2M (D) cell lines, respectively. The MGSTA-4 treatment at the concentration of 100 µM significantly decreased viability of the CMT-W1 (A) and CMT-W2 (B) cell lines. The MGSTA-2 treatment at the concentration of 100 µM significantly decreased viability of the CMT-W1 (A) cell line. The statistical analysis was performed using GraphPad Prism version 5.00 software (GraphPad Software, USA). One-way ANOVA followed by Dunnett’s post hoc comparisons were applied. p < 0.01 was marked as **.
(TIF)

Figure S2 **Dose-dependent inhibition of migration by MGSTA-6 in CMT-W1, CMT-W1M and CMT-W2 canine cancer cells evaluated in trans-well migration assay.** MGSTA-6 given at various concentrations ranging from 0.1 to 100 µM inhibited migration of canine cancer cell lines. The graphs present dose-dependent curves and IC_50_ values for each cell line. The experiment was performed three times. The statistical analysis was done using dose-dependent inhibition pattern (log{inhibitor}vs. response {three parameters}) with Prism version 5.00 software (GraphPad Software, USA).
(TIF)

Figure S3 **Representative confocal microscopy images of cytoskeletal protein F-actin and fascin1 in canine carcinoma cell lines.** The images demonstrated actin (green) an fascin1 (red) localization in the CMT-W1 (A), CMT-W1M (B) cell lines, in the control conditions (upper row) and after MGSTA-6 treatment (lower row). In control condition multiple filopodia protrusion was observed as well as stress fibers (arrowheads in upper row). In addition, expression of fascin1 strongly co-localized with F-actin (merge images in upper row). In contrast, cells treated with MGSTA-6 lost the stress fibers and presented more free-fascin1 protein (not associated with F-actin) in the central area (arrows in lower row). Furthermore CMT-W2 (B) cells formed lamellipodia at the edge (arrows in upper row), while after administration of MGSTA-6 formation of lammellipodia was inhibited. The CMT-2M (C) cells did not formed many filopodial protrusion and stress fibres, however after MGSTA-6 treatment the fascin1 was localized at the cell periphery (arrows at merge image in the lower row). Cells were visualized using the confocal laser scanning microscope FV-500 system at the magnification of x60, zoom 1.5 (A); 2.0 (B, C) (Olympus Optical Co, Hamburg, Germany). D. Quantification of fascin1 and F-actin co-localization at merge images, using computer-assisted image analyser (Olympus Microimage™ Image Analysis, software version 5.0 for windows, USA). The analysis revealed that administration of MGSTA-6 cause statistically significant reduction of co-localization of those two proteins in CMT-W1, CMT-W1M and CMT-W2M cells. There were no differences in that process in CMT-W2M cell. The colorimetric intensity of spots related to co-localization of both proteins was presented as Integrated Optical Density (IOD) ± SD. Five to ten pictures in each slide were analysed. The experiment was performed three times. The statistical analysis was done using Prism version 5.00 software (GraphPad Software, USA). The unpaired t-test was applied. p < 0.05 was marked as * p < 0.01 was marked as **.
(TIF)

Figure S4 **Expression of phospho-FSCN1(Ser39) in examined canine mammary cancer cell lines.** The intensity of red fluorescence related to fascin1 expression was assessed using computer-assisted image analyser (Olympus Microimage™ Image Analysis, software version 5.0 for windows, USA) and presented as Integrated Optical Density (IOD) ± SEM. The analysis revealed that in control conditions protein level of phospho-fascin1 is lower in CMT-W2M than in other cell lines, especially compared with CMT-W1M cells. The experiment was performed three times. Five to ten pictures in each slide were analysed. The statistical analysis was conducted using Prism version 5.00 software (GraphPad Software, USA). The unpaired t-test was applied. p < 0.05 was marked as *.
(TIF)
