# Supplementary figures and images for: Migrastatin Analogues Inhibit Canine Mammary Cancer Cell Migration and Invasion
Source: PLoS One. 2013 Oct 8;8(10):e76789. doi: 10.1371/journal.pone.0076789 (PMC3792885; doi:10.1371/journal.pone.0076789)

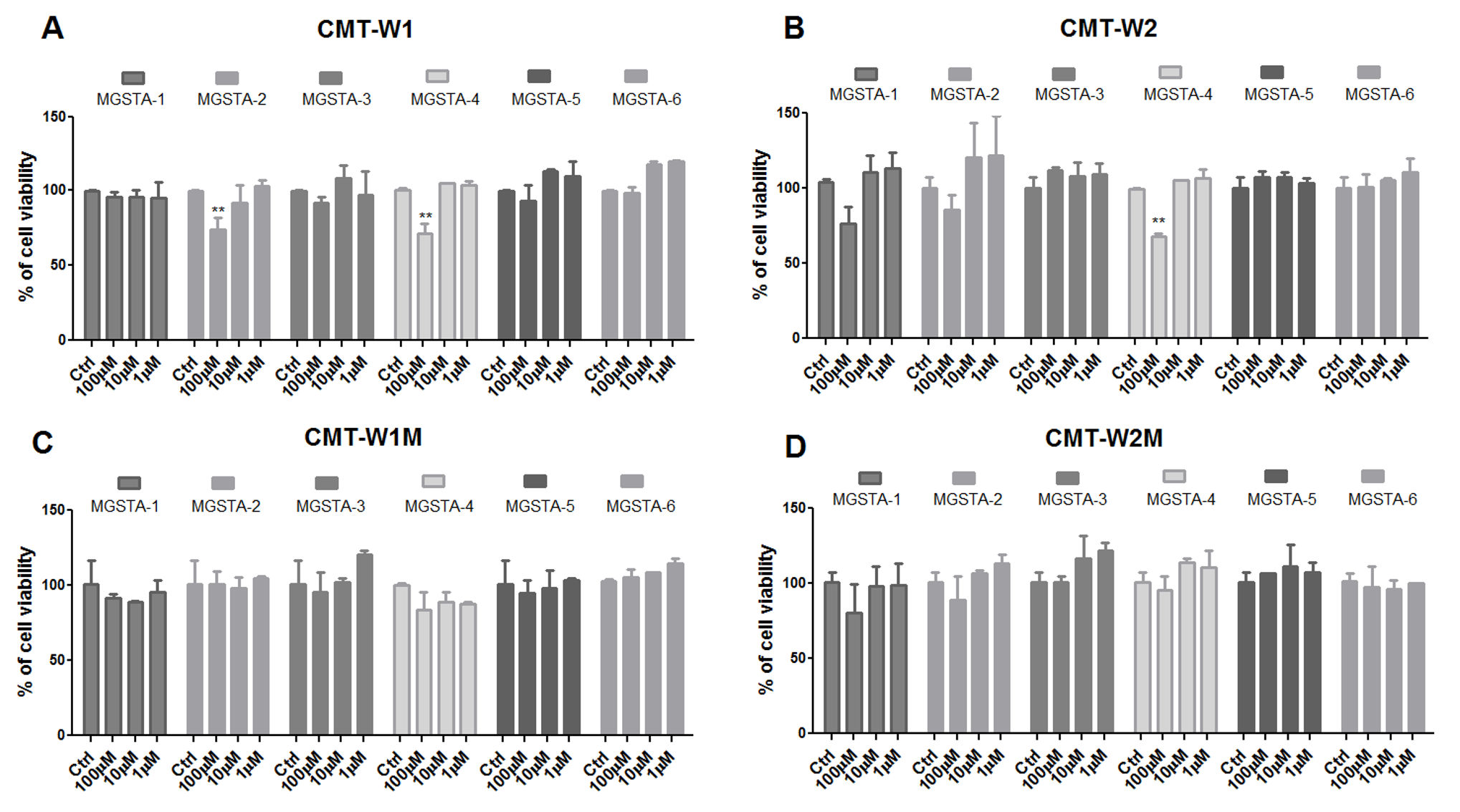

Supplement: Figure S1 — Influence of analogues of migrastatin on viability of canine mammary cancer cells. The canine mammary cancer cells viability was determined by MTT assay in control conditions (ctrl) and in the presence of six migrastatin analogues given at concentrations of 1, 10, or 100 µM for 24 h. Absorbance measured at 570 nm was converted to percentages (±S.D.). The experiment was conducted in n=9 repetitions. No significant differences were observed after incubation with MGSTA-1, 3 and 5-6 in CMT-W1 (A), CMT-W2 (B), CMT-W1M (C) and CMT-W2M (D) cell lines, respectively. The MGSTA-4 treatment at the concentration of 100 µM significantly decreased viability of the CMT-W1 (A) and CMT-W2 (B) cell lines. The MGSTA-2 treatment at the concentration of 100 µM significantly decreased viability of the CMT-W1 (A) cell line. The statistical analysis was performed using GraphPad Prism version 5.00 software (GraphPad Software, USA). One-way ANOVA followed by Dunnett’s post hoc comparisons were applied. p < 0.01 was marked as **. (TIF) [file pone.0076789.s001.tif]

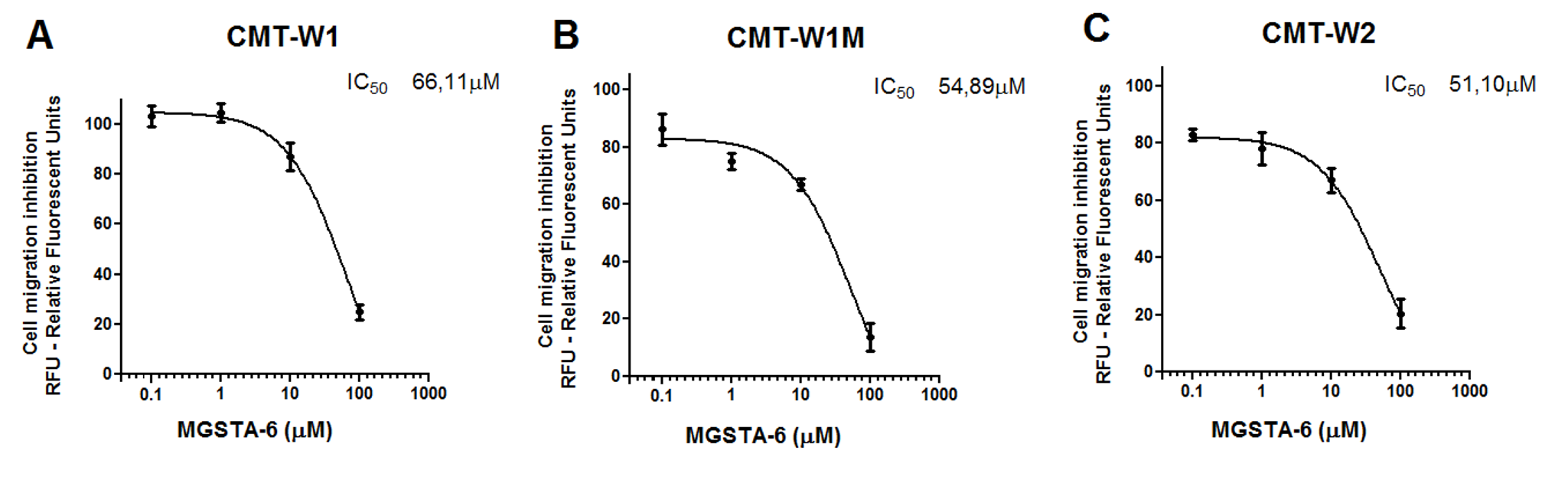

Supplement: Figure S2 — Dose-dependent inhibition of migration by MGSTA-6 in CMT-W1, CMT-W1M and CMT-W2 canine cancer cells evaluated in trans-well migration assay. MGSTA-6 given at various concentrations ranging from 0.1 to 100 µM inhibited migration of canine cancer cell lines. The graphs present dose-dependent curves and IC50 values for each cell line. The experiment was performed three times. The statistical analysis was done using dose-dependent inhibition pattern (log{inhibitor}vs. response {three parameters}) with Prism version 5.00 software (GraphPad Software, USA). (TIF) [file pone.0076789.s002.tif]

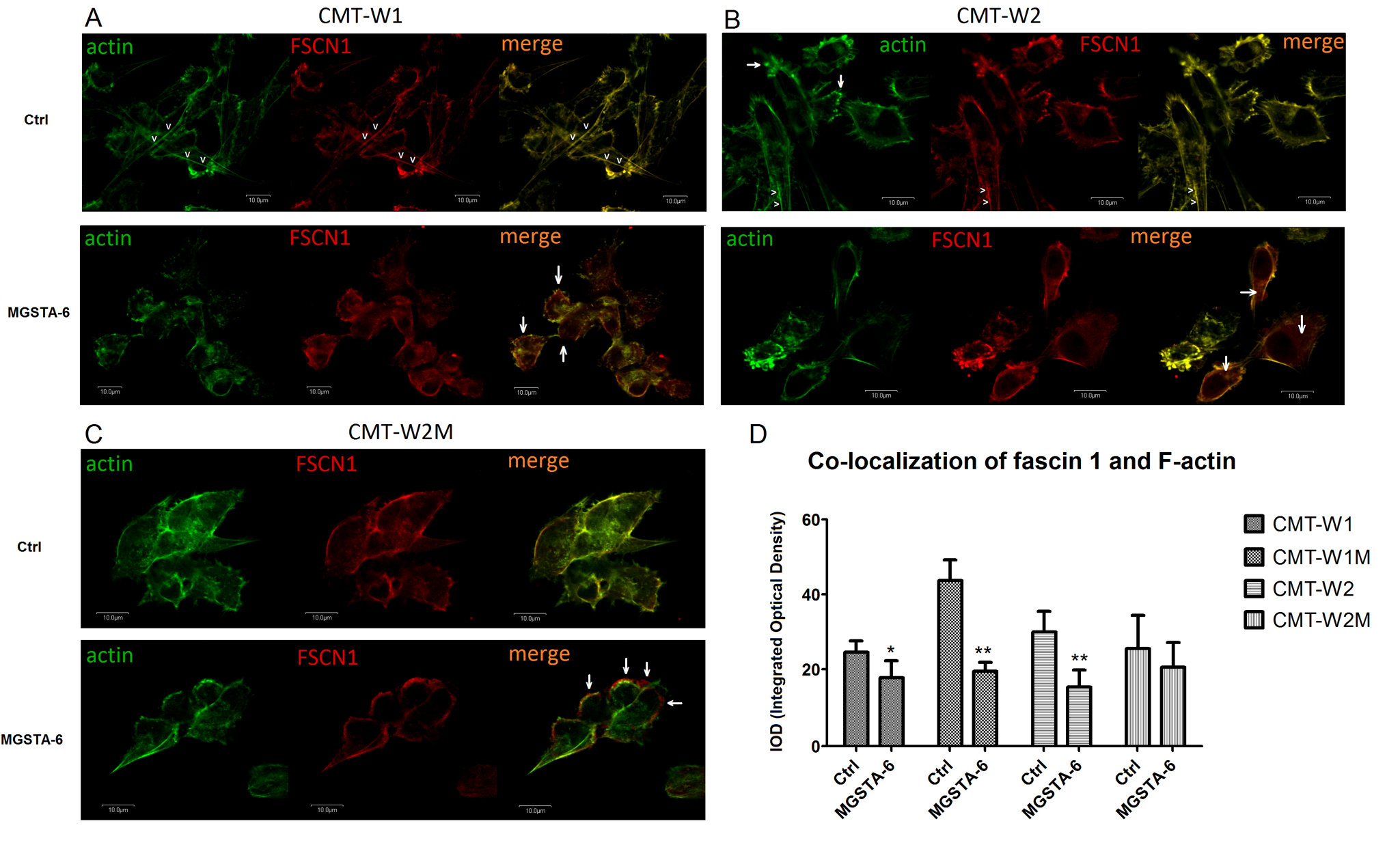

Supplement: Figure S3 — Representative confocal microscopy images of cytoskeletal protein F-actin and fascin1 in canine carcinoma cell lines. The images demonstrated actin (green) an fascin1 (red) localization in the CMT-W1 (A), CMT-W1M (B) cell lines, in the control conditions (upper row) and after MGSTA-6 treatment (lower row). In control condition multiple filopodia protrusion was observed as well as stress fibers (arrowheads in upper row). In addition, expression of fascin1 strongly co-localized with F-actin (merge images in upper row). In contrast, cells treated with MGSTA-6 lost the stress fibers and presented more free-fascin1 protein (not associated with F-actin) in the central area (arrows in lower row). Furthermore CMT-W2 (B) cells formed lamellipodia at the edge (arrows in upper row), while after administration of MGSTA-6 formation of lammellipodia was inhibited. The CMT-2M (C) cells did not formed many filopodial protrusion and stress fibres, however after MGSTA-6 treatment the fascin1 was localized at the cell periphery (arrows at merge image in the lower row). Cells were visualized using the confocal laser scanning microscope FV-500 system at the magnification of x60, zoom 1.5 (A); 2.0 (B, C) (Olympus Optical Co, Hamburg, Germany). D. Quantification of fascin1 and F-actin co-localization at merge images, using computer-assisted image analyser (Olympus Microimage™ Image Analysis, software version 5.0 for windows, USA). The analysis revealed that administration of MGSTA-6 cause statistically significant reduction of co-localization of those two proteins in CMT-W1, CMT-W1M and CMT-W2M cells. There were no differences in that process in CMT-W2M cell. The colorimetric intensity of spots related to co-localization of both proteins was presented as Integrated Optical Density (IOD) ± SD. Five to ten pictures in each slide were analysed. The experiment was performed three times. The statistical analysis was done using Prism version 5.00 software (GraphPad Software, USA). The unpaired [file pone.0076789.s003.tif]

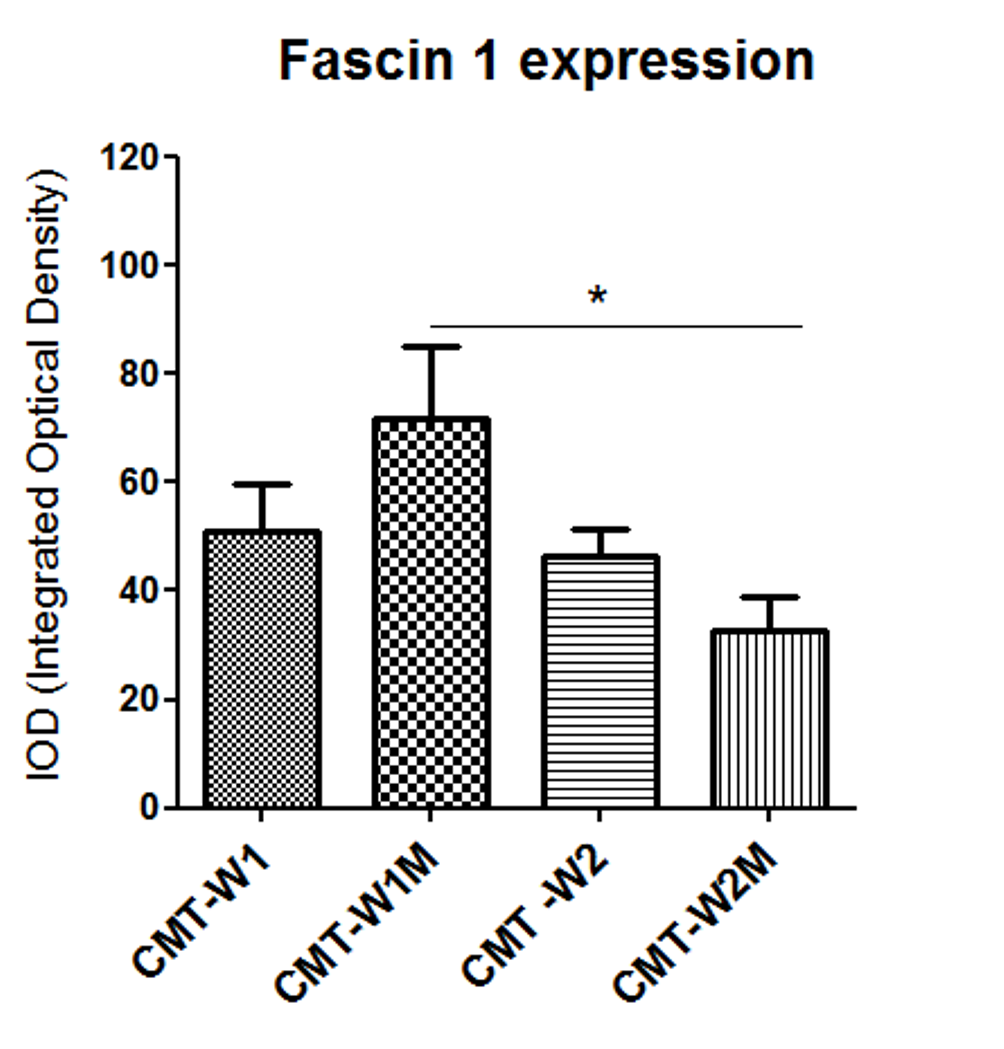

Supplement: Figure S4 — Expression of phospho-FSCN1(Ser39) in examined canine mammary cancer cell lines. The intensity of red fluorescence related to fascin1 expression was assessed using computer-assisted image analyser (Olympus Microimage™ Image Analysis, software version 5.0 for windows, USA) and presented as Integrated Optical Density (IOD) ± SEM. The analysis revealed that in control conditions protein level of phospho-fascin1 is lower in CMT-W2M than in other cell lines, especially compared with CMT-W1M cells. The experiment was performed three times. Five to ten pictures in each slide were analysed. The statistical analysis was conducted using Prism version 5.00 software (GraphPad Software, USA). The unpaired t-test was applied. p < 0.05 was marked as *. (TIF) [file pone.0076789.s004.tif]
